# Epidemiology, Prognosis, and Evolution of Management of Septic Shock in a French Intensive Care Unit: A Five Years Survey

**DOI:** 10.1155/2010/436427

**Published:** 2010-06-17

**Authors:** Nicolas Boussekey, Juliette Cantrel, Lise Dorchin Debrabant, Joachim Langlois, Patick Devos, Agnes Meybeck, Arnaud Chiche, Hugues Georges, Olivier Leroy

**Affiliations:** Intensive Care and Infectious Disease Unit, Tourcoing Hospital, University of Lille, 135, rue du Président Coty, BP 619, 59208 Tourcoing, France

## Abstract

*Purpose*. To evaluate the epidemiology, prognosis, and management of septic shock patients hospitalized in our intensive care unit (ICU). *Materiel and Methods*. Five-year monocenter observational study including 320 patients. *Results*. ICU mortality was 54.4%. Independent mortality risk factors were mechanical ventilation (OR = 4.97), Simplify Acute Physiology Score (SAPS) II > 60 (OR = 4.28), chronic alcoholism (OR = 3.38), age >65 years (OR = 2.65), prothrombin ratio <40% (OR = 2.37), and PaO_2_/FiO_2_ ratio <150 (OR = 1.91). These six mortality risk factors recovered allow screening immediately septic shock patients with a high mortality risk. Morbidity improved with time (diminution of septic shock complications, increase of the number of days alive free from mechanical ventilation and vasopressors on day 28), concomitant to an evolution of the management (earlier institution of all replacement and medical therapies and more initial volume expansion). There was no difference in mortality. *Conclusion*. Our study confirms a high mortality rate in septic shock patients despite a new approach of treatment.

## 1. Introduction

A better pathophysiologic knowledge and the apparition of international recommendations allowed an improvement of septic shock's prognosis, but mortality still remains above 50% [1, 2]. Numerous factors are associated to mortality in septic shock. Early recognition of these factors can help to identify the most critical situations and to provoke a more aggressive resuscitation. We realized an observational study including all the patients suffering from septic shock and hospitalized in our intensive care unit (ICU) from 2003 to 2007. Beyond an epidemiologic analysis, we wanted to identify mortality risk factors and evolution of septic shock management in our ICU.

## 2. Material and Methods

### 2.1. Inclusion Criteria and Study Aims

We included all the patients suffering from septic shock and hospitalized in the ICU of Tourcoing hospital, France, between January 2003 and January 2008. Our study had 3 aims: an epidemiologic record, the identification of independent mortality risk factors, and the evaluation of management and prognosis comparing 2 periods, before and after the publication of the Surviving Sepsis Campaign (SSC) international guidelines in December 2004 [3].

### 2.2. Data Collection and Definitions

#### 2.2.1. On Admission

For all patients, the following characteristics were prospectively collected on ICU admission: age, gender, underlying clinical conditions, severity of illness, and vital sign abnormalities. Severity of illness was assessed by the Simplified Acute Physiology Score (SAPS) II [4] and the Sepsis-related Organ Failure Assessment (SOFA) score [5]. We estimated an organ dysfunction if the score for this organ was superior to 3. Septic shock was defined as a sustained (≥1 h) decrease in the systolic blood pressure of at least 40 mmHg from baseline or a resultant systolic blood pressure <90 mmHg after adequate fluid replacement and in the absence of any antihypertensive drug [6]. We recorded the community or hospital-acquired origin of the infection, bacteriological data and if the patient presented coinfection (more than one pathologic bacteria recovered). We also noted the delay from the hospital admission, from the onset of the sepsis and the septic shock, to the admission in the ICU. We recorded all the bacteriological samples as well as the initial antibiotic therapy. Antibiotic therapy was defined as adapted (if it followed recommendations), and adequate (if it comported at least one antibiotic active in vitro on the causal bacteria). Delay from the onset of the sepsis to the start of the antibiotic treatment was also noted. 

Volume expansion with hydroxyethylstarches (HES), cristalloids, 4% albumin and packed red blood cells was quantified at 6 and 24 hours after the onset of the sepsis as well as vasopressor use. We also recorded the use of activated protein C, hydrocortisone, intensive insulin therapy, type and amount of nutrition, need for mechanical ventilation or renal replacement therapy (RRT). We also noted the time from the beginning of vasopressors and the prescription of hydrocortisone, nutrition, ventilation, and RRT.

#### 2.2.2. Evolution

We recorded if the patients presented a recurrence of shock (need for vasopressor after 8 hours weaning). During the ICU stay, occurrence of complications was recorded. We distinguished infection-related complications (acute respiratory distress syndrome (ARDS), acute renal failure, disseminated intravascular coagulation, and acute hepatic failure) and hospital-acquired infections. Finally, we recorded the number of days alive free from mechanical ventilation, vasopressors, and RRT on day 28. Mortality was evaluated on day 28 and at ICU discharge.

### 2.3. Statistical Analysis

To determine the factors associated with mortality, we realized a logistic regression analysis. First, we performed a monovariate analysis, and then pooled the significant parameters (*P* < .2) in a multivariate analysis with the estimate of the Odds Ratio (OR). For multivariate analysis, an area under the curve was used to determine the cut-off of continuous variables. Comparisons between groups were performed using Chi-square test or Fisher's exact test for categorical parameters. Continuous variables were analysed using Wilcoxon's test. Differences between groups were considered to be significant for variables yielding a *P*  value < .05. All analyses were performed using the SAS Software, V8.2.

## 3. Results

### 3.1. Baseline Characteristics on Admission

Between January 2003 and January 2008, 320 patients were hospitalized in our ICU for septic shock. Sixty three percent of the population was male, mean age was 65 ± 15.3 years, SAPS II and SOFA score were 62.3 ± 20.1 and 11.3 ± 3.2, respectively. The major comorbidity was chronic alcoholism (26.8%), followed by chronic obstructive pulmonary disease (COPD (25.2%)), diabete mellitus (21.8%), and chronic cardiac failure (18%). Associated organ failures were respiratory (83.6%), neurologic (35.7%), renal (26.3%), coagulation (15.2%), and hepatic (3.5%). Mean times from the hospital admission, the onset of the sepsis and the septic shock to ICU admission were 3.8 ± 7.5 days, 16.7 ± 28.8 hours, and 2.4 ± 4.8 hours, respectively.

### 3.2. Bacteriology and Antibiotic Therapy

The sepsis was community-acquired in 64% of the patients. Infectious site was predominantly respiratory (48.4%), followed by intraabdominal (20%) and urinary (8.7%). The infection was bacteriologically documented in 59% of the patients, with 33% of positive blood cultures. 20.4% of the patients presented coinfections. Causative pathogens were Gram-positive cocci in 45.3% of the positive cultures, mostly represented by *Streptococcus pneumoniae* (17.4%) and *Staphylococcus aureus* methicillin-sensitive (11.6%), and methicillin-resistant (4.2%). Gram-negative bacilli were found in 43.2%: *Escherichia coli* (15.8%), *Klebsiella* (5.3%), and *Pseudomonas aeruginosa* ticarcillin-sensitive (4.7%) and ticarcillin-resistant (3.7%). Anaerobes bacteria represented 3.7% and Candida 2.6% of the cases. Mean time from the onset of the sepsis to the introduction of antibiotics was 9.8 ± 18.7 hours. It was adapted in 97% and adequate in 88% of the patients.

### 3.3. Management

Mean plasma volume expansion during the 24 hours following the sepsis was 1160 ± 793 mL for HES and 2194 ± 1720 mL for cristalloids. The most used vasopressor agent was norepinephrine in 74%, followed by dopamine (42%), dobutamine (26%), and epinephrine (16%). Only 7% of the patients received activated protein C. Hydrocortisone was prescribed in 84% of the population, 7.9 ± 12 hours after the onset of shock. Mechanical ventilation was needed in 88%; noninvasive ventilation was performed in 13% of the patients. Thirty one percent of the patients needed RRT.

### 3.4. Prognosis on Day 28

Recurrence of shock was noted in 24% of the population, complications of septic shock in 28.2% and hospital-acquired infections in 25.7% with 32% of multiresistant bacteria. ICU mortality was 54.4%, day 28 mortality 51.2%. Time alive free from ICU, mechanical ventilation, vasopressors, and RRT on day 28 was 5.6 ± 8.8 days, 5.3 ± 8.4 days, 10.1 ± 10.5 days and 9 ± 11.2 days, respectively.

### 3.5. Independent Mortality Risk Factors

For all the collected variables, we realized a bivariate analysis (Table 1), and then all the significant parameters were entered in a multivariate analysis. We found 6 independent variables associated with mortality: need for mechanical ventilation, SAPS II >60, chronic alcoholism, age >65 years, prothrombin ratio (PR) <40% and PaO_2_/FiO_2 _<150 (Table 2).

### 3.6. Comparison of the 2003-2004 and 2005–2007 Periods

The 2003-2004 period represented 41.9% of the population. The 2 groups were homogeneous for baseline characteristics except more chronic cardiac failures (27.8% versus 10.8%, *P* = .001) and a lower SOFA score (10.9 ± 3.2 versus 11.6 ± 3.2, *P* = .0338) during the 2003-2004 period. In contrast, the management of the 2 groups was very different: in the second period, norepinephrine became the vasoactive agent of choice (*P* < .0001) instead of dopamine and dobutamine, initial plasma volume expansion was greater (*P* = .0005), patients received more intensive insulin therapy (*P* < .0001), and the delays of intubation (*P* = .01), antibiotic therapy (*P* = .0037), initiation of hydrocortisone (*P* = .0001), RRT (*P* = .002), and nutrition (*P* = .0389) were shortened (Table 3).

Prognosis variables were also different between the 2 periods. We found during the period 2005–2007 an increase in the number of days alive free from mechanical ventilation (*P* = .0207) and vasopressors (*P* = .0021) on day 28, and a decrease in septic shock complications (*P* < .0001). However, there was no difference in mortality (*P* = .79). All the significant differences concerning the prognosis are presented Table 4.

## 4. Discussion

### 4.1. Epidemiology and Prognosis

Mean age was 65 years and there was a predominance of male. Three French epidemiologic studies found a similar repartition [1, 2, 7]. In contrast, our population was particularly severely ill; mean SAPS II and SOFA scores on admission were 62 and 11, respectively, compared to 58 and 9 in these studies [1, 2]. The infection was bacteriologically proven in 59% of the patients, as in the other studies [2, 8, 9]. Literature data also reveal a change in the origin of the sepsis with a decrease of intraabdominal and Gram-negative bacilli infections replaced by pulmonary and Gram-positive cocci infections [10–12]. It is in accordance with our results: half of the infections was coming from the respiratory tract and was caused by Gram-positive cocci. Annane, realizing a multicenter epidemiologic study between 1993 to 2000 found a 60.1% mortality rate, with an improvement from 1993 (62.1%) to 2000 (55.6%) [2]. This improvement seems to be related to an earlier and more aggressive management and an improvement in specific anti-infectious therapies [13–15]. ICU mortality in our study was 54.4%. Most other published studies found a 35% mortality rate, but patients with severe sepsis were also included [1, 10, 16–18]. 

We recovered six independent mortality risk factors: mechanical ventilation, SAPS II >60, chronic alcoholism, age >65 years, PR <40%, and PaO_2_/FiO_2_ ratio <150. These factors can easily be available on admission and allow screening immediately a group of patients with a high mortality risk. Our results confirm the classic risk factors associated with mortality recovered in the literature [1, 2, 7, 10]. Specifically regarding chronic alcoholism, numerous studies report its association with the frequency of infections [19, 20], but an important role in mortality in the absence of hepatic failure is not well known, excepted in the study by O'Brien [21] who found an increase in mortality in ICU patients suffering from chronic alcoholism, and particularly when patients developed severe sepsis and septic shock. Its deleterious effect was probably revealed by the high prevalence of alcoholism in our population (26.8%).

Most shocking is the absence of signification of antibiotic therapy. Numerous studies [9, 22, 23] showed an impairment of the prognosis when antibiotic therapy was delayed. In particular, Kumar found a 7.6% increased mortality for each hour delay of antibiotics [24]. Mean time for antibiotic therapy in our study was very long (9.77 hours), but it was calculated from the onset of the sepsis and not septic shock. Adequate antibiotic therapy improves the prognosis of patients suffering from septic shock. In our study, antibiotic therapy was adapted in 97% of the patients and adequate in 88%. These results are excellent, compared, for example, to a 2003 american study who found 23% of nonadapted antibiotic therapy, responsible of an increase in mortality [9]. These important percentages probably explain the absence of signification of antibiotics in our study.

### 4.2. Comparison of 2003-2004 and 2005–2007 Periods

In spite of a higher SOFA score during the 2005–2007 period, the mortality remained the same but some variables improved (diminution of septic shock complications, increase of the number of days alive free from mechanical ventilation and vasopressors on day 28). We tried to find if the evolution in the management of our patients could explain these differences. The first hours are critical in septic shock and the major difference in the management between the 2 periods was the rapidity to institute all medical or replacement therapies. Likewise, initial plasma volume expansion was greater in the last period. Nevertheless, these volumes of fluids (about 3.5 litres in 24 hours) are far below others studies where patients received nearly 5 or 6 litres [1, 3, 7, 11, 12, 14, 23]. The aggressive 〈〈  early  goal  directed  therapy  〉〉 [14] is recommended by the SSC [3], because of its beneficial effect on mortality. Even with an increase in plasma volume expansion between the 2 periods, it remains probably too low. This is well explained by the difficulty to translate prospective controlled studies in daily clinical practice. Moreover, we collected the data from the onset of the sepsis when patients were often hospitalized in other units and physicians who are not intensivists are not sensitized to the importance of an aggressive volume expansion.

Regarding the hemodynamic support, norepinephrine became the vasopressor of choice in replacement of dopamine. Likewise, the dobutamine was used very often before 2005 and its indication was restricted after. This is conforming to the recommendations of the SSC [3]. Activated protein C is a controversial treatment of septic shock and is not a high grade recommendation in the 2008 SSC [25]. Waiting for the results of ongoing studies, we do not often use this molecule (4.5% and 8.1% of the patients during the 2 periods), so we cannot evaluate it in our study. Hydrocortisone was largely used during the 2 periods (80.6% and 87.1%), following the study published by Annane in 2002 [26]. Finally, the 2004 recommendations [3] have contributed to introduce more intensive insulin therapy in our ICU. Van den Berghe et al. [27] have demonstrated in 2001 a reduction of mortality in surgical patients when maintaining glycaemia between 0.8 to 1.1 g/l with a decrease in multiorgan failures secondary to septic shock. The same team [28] did not confirm their results in a population of medical patients and the study recently published by Arabi do not support this intensive strategy [29]. In our study, we did not find deleterious effect of insulin, but we did not recorded hypoglycaemia.

## 5. Conclusion

The six mortality risk factors recovered in the multivariate analysis can easily be available on admission and allow screening immediately a group of patients with a high mortality risk. Concerning the evolution of the management during these 5 years, even if it is difficult in usual practice to be completely in accordance with recommendations, we remark an earlier introduction of numerous replacement and medical therapies as well as a more initial aggressive volume expansion. This probably explains the improvement in morbidity, whereas mortality did not change.

## Figures and Tables

**Table 1:** Mortality risk factors in septic shock patients: monovariate analysis COPD: chronic obstructive pulmonary disease, SAPS: simplify acute physiology score, SOFA: sepsis-related organ failure assessment score, ICU: intensive care unit, RRT: renal replacement therapy, prothrombin ratio (PR).

	Survivors 146 (45.6)	Deceased174 (54.4)	*P*
*Comorbidities * *n*(*%) *			
Chronic cardiac failure	21(14.4)	36(21)	.1233
Diabete mellitus	29(19.9)	40(23.4)	.4479
COPD	35(23.9)	45(26.3)	.6321
Chronic liver failure	11(7.5)	24(14)	.0656
Chronic alcoolism	28(19.1)	57(33.3)	.0046
Chronic renal failure	6(4.1)	10(5.8)	.4810
Non hematologic malignancy	16(10.9)	20(11.6)	.8367
Hematologic malignancy	16(10.9)	16(9.3)	.6369
Immunosuppression	25(17.1)	24(14)	.4484
*Clinical presentation*			
Male sex *n*(%)	92(63)	111(63.8)	.8853
Age (years) (mean ± SD)	61.6 ± 15.5	68.5 ± 14.3	<.0001
Lactate (meq/l) (mean ± SD)	3 ± 2.2	4.9 ± 4	<.0001
Platelet count (1000/mm³) (mean ± SD)	203 ± 144	198 ± 143	.7813
Creatinine (mg/l) (mean ± SD)	21.6 ± 15.4	22.7 ± 12.3	.4825
Bilirubine (mg/l) (mean ± SD)	14.2 ± 13.5	20.6 ± 27.4	.0157
PR (%) (mean ± SD)	58.6 ± 19.8	49.6 ± 20.5	<.0001
pH (mean ± SD)	7.3 ± 0.1	7.2 ± 0.1	.0007
PaO_2_/FiO_2_ (mean ± SD)	190 ± 103	154 ± 101	.0021
SAPS II (mean ± SD)	52.7 ± 13.9	70.3 ± 20.9	<.0001
SOFA score (mean ± SD)	10.6 ± 3	11.9 ± 3.1	.0002
Respiratory failure *n*(%)	123(84.2)	153(88.4)	.2746
Renal failure *n*(%)	30(20.5)	53(31.1)	.0323
Coagulation failure *n*(%)	21(14.3)	29(17.2)	.4869
Hepatic failure *n*(%)	1(0.7)	10(6)	.0108
Neurologic failure *n*(%)	40(28.5)	70(41.6)	.0169
*Management*			
Time from shock to ICU admission (hours) (mean ± SD)	2.5 ± 5.6	2.2 ± 4	.6034
Adapted antibiotic therapy *n*(%) (mean ± SD)	138(97.8)	154(96.8)	.7269
Time from sepsis to adapted antibiotic therapy (hours) (mean ± SD)	8 ± 18.2	11.2 ± 19	.1426
Adequate antibiotic therapy *n*(%)	89(92.7)	76(83.5)	.0512
Time from sepsis to adequate antibiotic therapy (hours) (mean ± SD)	10.3 ± 23.3	15.4 ± 25.7	.1698
Mechanical ventilation *n*(%)	116(79.4)	166(95.9)	<.0001
Noninvasive ventilation *n*(%)	12(8.6)	27(15.6)	.0614
Time from shock to intubation (hours) (mean ± SD)	2.5 ± 6.4	3.3 ± 9.8	.4050
RRT *n*(%)	41(28)	57(33.1)	.3304
Time from shock to RRT (hours) (mean ± SD)	29 ± 38.7	29.7 ± 33.4	.9216
Cristalloid volume expansion at 6 hours of sepsis (mL) (mean ± SD)	1302 ± 1123	974 ± 1127	.0135
Cristalloid volume expansion at 24 hours of sepsis (mL) (mean ± SD)	2629 ± 1748	1842 ± 1618	<.0001
Hydrocortisone *n*(%)	123(84.2)	147(84.4)	.9538
Time from shock to initiation of hydrocortisone (hours) (mean ± SD)	8.2 ± 13	7.4 ± 10.9	.5985
Activated proteine C *n*(%)	13(8.9)	8(4.6)	.1246
Intensive insulin therapy *n*(%)	94(64.3)	86(49.4)	.0072
Enteral nutrition *n*(%)	101(70.6)	67(51.1)	.0009
Parenteral nutrition *n*(%)	39(27.2)	29(22.1)	.3256
Time from shock to initiation of nutrition (days) (mean ± SD)	2.5 ± 1.3	2.4 ± 1.1	.7918

**Table 2:** Independent mortality risk factors in septic shock patients: multivariate analysis SAPS: simplify acute physiology score, prothrombin ratio (PR).

	Odds ratio	Confidence interval	*P*
Mechanical ventilation	4.97	[1.79–13.75]	.0021
SAPS II >60	4.28	[2.51–7.13]	.0001
Chronic alcoholism	3.38	[1.75–6.52]	.0003
Age >65 years	2.65	[1.47–4.79]	.0012
PR <40%	2.37	[1.26–4.45]	.0074
PaO_2_/FiO_2_<150	1.91	[1.15–3.26]	.00183

**Table 3:** Baseline characteristics and management of septic shock patients: significant differences between the 2003-2004 and 2005–2007 periods. COPD: chronic obstructive pulmonary disease, SAPS: simplify acute physiology score, SOFA: sepsis-related organ failure assessment score, ICU: intensive care unit, RRT: renal replacement therapy, prothrombin ratio (PR).

	2003-2004	2005–2007	*P*
*Comorbidities n(%)*			
Chronic cardiac failure	37(27.8)	20(10.8)	.001
Diabete mellitus	34(25.6)	35(19)	.16
COPD	39(29.3)	41(22.3)	.1544
Chronic liver failure	19(14.3)	16(8.7)	.1171
Chronic alcoolism	(24.8)	(28.3)	.4939
Chronic renal failure	8(6)	8(4.3)	.5034
Non hematologic malignancy	8(6)	28(15.2)	.8367
Hematologic malignancy	18(13.5)	14(7.6)	.084
Immunosuppression	18(13.5)	31(16.8)	.4205
*Clinical presentation*			
Male sex *n*(%)	82(61.2)	121(65)	.4794
Age (years) (mean ± SD)	63.9 ± 15.2	66.5 ± 15.3	.1374
Lactate (meq/l) (mean ± SD)	4.6 ± 4.1	3.7 ± 2.9	.0993
Platelet count (1000/mm³) (mean ± SD)	180 ± 1410	215 ± 144	.0345
Creatinine (mg/l) (mean ± SD)	22.4 ± 14.6	22.1 ± 13.3	.8113
Bilirubine (mg/l) (mean ± SD)	20.8 ± 21.1	15.4 ± 22.4	.0425
PR (%) (mean ± SD)	52.1 ± 21.5	55.1 ± 20.1	.2117
pH (mean ± SD)	7.29 ± 0.14	7.29 ± 0.13	.7409
PaO_2_/FiO_2_ (mean ± SD)	161.1 ± 96.4	177.9 ± 107.9	.1649
SAPS II (mean ± SD)	60.8 ± 20.1	63.4 ± 20.1	.259
SOFA score (mean ± SD)	10.9 ± 3.2	11.6 ± 3.2	.0338
*Management*			
Mechanical ventilation *n*(%)	(85.1)	(90.8)	.1143
Norepinephrine *n*(%)	79(59)	158(85.4)	<.0001
Dobutamine *n*(%)	51(38.1)	32(17.4)	<.0001
Dopamine *n*(%)	91(67.9)	43(23.4)	<.001
Intensive insulin therapy *n*(%)	17(12.7)	163(87.6)	<.0001
Time from sepsis to adapted antibiotic treatment (hours) (mean ± SD)	13.9 ± 18.7	7.2 ± 18.3	.0037
Cristalloid volume expansion at 6 hours of sepsis (mean ± SD)	816 ± 1038	1323 ± 1155	.0001
Cristalloid volume expansion at 24 hours of sepsis (mean ± SD)	1773 ± 1674	2479 ± 1696	.0005
Time from shock to initiation of hydrocortisone (hours) (mean ± SD)	11.3 ± 14	5.6 ± 9.8	.0001
Time from shock to intubation (hours) (mean ± SD)	4.6 ± 11.9	1.9 ± 5.2	.0105
Time from shock to RRT (hours) (mean ± SD)	43.6 ± 42	20.8 ± 28.1	.002
Time from shock to initiation of nutrition (days) (mean ± SD)	2.7 ± 1.4	2.3 ± 1.1	.0389

**Table 4:** Prognosis of septic shock patients: comparison between the 2003-2004 and 2005–2007 periods. ICU: intensive care unit, RRT: renal replacement therapy.

	2003-2004	2005–2007	*P*
Septic shock complications *n*(%)	60(46.5)	29(15.6)	<.0001
Number of days alive free from mechanical ventilation on day 28 (mean ± SD)	3.9 ± 6.9	6.2 ± 9.1	.0207
Number of days alive free from vasopressors on day 28 (mean ± SD)	8 ± 9.6	11.6 ± 10.9	.0021
Number of days alive free from RRT on day 28 (mean ± SD)	8.6 ± 12.1	9.2 ± 10.7	.76
Patients discharged from ICU on day 28 *n*(%)	46(34.6)	69(37.1)	.6452
ICU death *n*(%)	74(55.2)	100(53.8)	.7958

## References

[1] Brun-Buisson C, Meshaka P, Pinton P, Vallet B (2004). EPISEPSIS: a reappraisal of the epidemiology and outcome of severe sepsis in French intensive care units. *Intensive Care Medicine*.

[2] Annane D, Aegerter P, Jars-Guincestre MC, Guidet B (2003). Current epidemiology of septic shock: the CUB-Réa network. *American Journal of Respiratory and Critical Care Medicine*.

[3] Dellinger RP, Carlet JM, Masur H (2004). Surviving Sepsis Campaign guidelines for management of severe sepsis and septic shock. *Critical Care Medicine*.

[4] Le Gall J-R, Lemeshow S, Saulnier F (1993). A new Simplified Acute Physiology Score (SAPS II) based on a European/North American multicenter study. *Journal of the American Medical Association*.

[5] Vincent J-L, Moreno R, Takala J (1996). The SOFA (Sepsis-related Organ Failure Assessment) score to describe organ dysfunction/failure. *Intensive Care Medicine*.

[6] Bone RC, Balk RA, Cerra FB (1992). Definitions for sepsis and organ failure and guidelines for the use of innovative therapies in sepsis. *Chest*.

[7] Brun-Buisson C, Doyon F, Carlet J (1995). Incidence, risk factors, and outcome of severe sepsis and septic shock in adults: a multicenter prospective study in intensive care units. *Journal of the American Medical Association*.

[8] Annane PD, Bellissant PE, Cavaillon J-M (2005). Septic shock. *Lancet*.

[9] Harbarth S, Garbino J, Pugin J, Romand JA, Lew D, Pittet D (2003). Inappropriate initial antimicrobial therapy and its effect on survival in a clinical trial of immunomodulating therapy for severe sepsis. *American Journal of Medicine*.

[10] Angus DC, Linde-Zwirble WT, Lidicker J, Clermont G, Carcillo J, Pinsky MR (2001). Epidemiology of severe sepsis in the United States: analysis of incidence, outcome, and associated costs of care. *Critical Care Medicine*.

[11] Russell JA (2008). The current management of septic shock. *Minerva Medica*.

[12] Friedman G, Silva E, Vincent J-L (1998). Has the mortality of septic shock changed with time?. *Critical Care Medicine*.

[13] Nobre V, Sarasin FP, Pugin J (2007). Prompt antibiotic administration and goal-directed hemodynamic support in patients with severe sepsis and septic shock. *Current Opinion in Critical Care*.

[14] Rivers E, Nguyen B, Havstad S (2001). Early goal-directed therapy in the treatment of severe sepsis and septic shock. *The New England Journal of Medicine*.

[15] Iscimen R, Cartin-Ceba R, Yilmaz M (2008). Risk factors for the development of acute lung injury in patients with septic shock: an observational cohort study. *Critical Care Medicine*.

[16] Alberti C, Brun-Buisson C, Burchardi H (2002). Epidemiology of sepsis and infection in ICU patients from an international multicentre cohort study. *Intensive Care Medicine*.

[17] Vincent J-L, Sakr Y, Sprung CL (2006). Sepsis in European intensive care units: results of the SOAP study. *Critical Care Medicine*.

[18] Blanco J, Muriel-Bombín A, Sagredo V (2008). Incidence, organ dysfunction and mortality in severe sepsis: a Spanish multicentre study. *Critical Care*.

[19] Díaz LE, Montero A, González-Gross M, Vallejo AI, Romeo J, Marcos A (2002). Influence of alcohol consumption on immunological status: a review. *European Journal of Clinical Nutrition*.

[20] von Dossow V, Schilling C, Beller S (2004). Altered immune parameters in chronic alcoholic patients at the onset of infection and of septic shock. *Critical Care*.

[21] O’Brien JM, Lu B, Ali NA (2007). Alcohol dependence is independently associated with sepsis, septic shock, and hospital mortality among adult intensive care unit patients. *Critical Care Medicine*.

[22] Gerlach H, Keh D (2003). Recent progress in sepsis epidemiology—have we learned enough?. *Critical Care*.

[23] Bochud P-Y, Bonten M, Marchetti O, Calandra T (2004). Antimicrobial therapy for patients with severe sepsis and septic shock: an evidence-based review. *Critical Care Medicine*.

[24] Kumar A, Roberts D, Wood KE (2006). Duration of hypotension before initiation of effective antimicrobial therapy is the critical determinant of survival in human septic shock. *Critical Care Medicine*.

[25] Dellinger RP, Levy MM, Carlet JM (2008). Surviving Sepsis Campaign: international guidelines for management of severe sepsis and septic shock: 2008. *Intensive Care Medicine*.

[26] Annane D, Sébille V, Charpentier C (2002). Effect of treatment with low doses of hydrocortisone and fludrocortisone on mortality in patients with septic shock. *Journal of the American Medical Association*.

[27] Van Den Berghe G, Wouters P, Weekers F (2001). Intensive insulin therapy in critically ill patients. *The New England Journal of Medicine*.

[28] van den Berghe G, Wilmer A, Hermans G (2006). Intensive insulin therapy in the medical ICU. *The New England Journal of Medicine*.

[29] Arabi YM, Dabbagh OC, Tamim HM (2008). Intensive versus conventional insulin therapy: a randomized controlled trial in medical and surgical critically ill patients. *Critical Care Medicine*.

